# Grape-Seed Procyanidin Extract (GSPE) Seasonal-Dependent Modulation of Glucose and Lipid Metabolism in the Liver of Healthy F344 Rats

**DOI:** 10.3390/biom12060839

**Published:** 2022-06-17

**Authors:** Romina M. Rodríguez, Marina Colom-Pellicer, Jordi Blanco, Enrique Calvo, Gerard Aragonès, Miquel Mulero

**Affiliations:** 1 Nutrigenomics Research Group, Department of Biochemistry and Biotechnology, Campus Sescelades, Universitat Rovira i Virgili (URV), 43007 Tarragona, Spain; rominamariel.rodriguez@urv.cat (R.M.R.); marina.colom@urv.cat (M.C.-P.); enrique.calvo@urv.cat (E.C.); gerard.aragones@urv.cat (G.A.); 2 Research in Neurobehavior and Health (NEUROLAB), Laboratory of Toxicology and Environmental Health, Physiology Unit, Institute of Health Research Pere Virgili (IISPV), School of Medicine, Universitat Rovira i Virgili (URV), 43202 Tarragona, Spain; jordi.blanco@urv.cat

**Keywords:** photoperiod, seasonal, GSPE, AMPK, clock genes, liver

## Abstract

Seasonality is gaining attention in the modulation of some physiological and metabolic functions in mammals. Furthermore, the consumption of natural compounds, such as GSPE, is steadily increasing. Consequently, in order to study the interaction of seasonal variations in day length over natural compounds’ molecular effects, we carried out an animal study using photo-sensitive rats which were chronically exposed for 9 weeks to three photoperiods (L6, L18, and L12) in order to mimic the day length of different seasons (winter/summer/and autumn-spring). In parallel, animals were also treated either with GSPE 25 (mg/kg) or vehicle (VH) for 4 weeks. Interestingly, a seasonal-dependent GSPE modulation on the hepatic glucose and lipid metabolism was observed. For example, some metabolic genes from the liver (SREBP-1c, *Gk*, *Acacα*) changed their expression due to seasonality. Furthermore, the metabolomic results also indicated a seasonal influence on the GSPE effects associated with glucose-6-phosphate, D-glucose, and D-ribose, among others. These differential effects, which were also reflected in some plasmatic parameters (i.e., glucose and triglycerides) and hormones (corticosterone and melatonin), were also associated with significant changes in the expression of several hepatic circadian clock genes (*Bmal1*, *Cry1*, and *Nr1d1*) and ER stress genes (*Atf6*, *Grp78*, and *Chop*). Our results point out the importance of circannual rhythms in regulating metabolic homeostasis and suggest that seasonal variations (long or short photoperiods) affect hepatic metabolism in rats. Furthermore, they suggest that procyanidin consumption could be useful for the modulation of the photoperiod-dependent changes on glucose and lipid metabolism, whose alterations could be related to metabolic diseases (e.g., diabetes, obesity, and cardiovascular disease). Furthermore, even though the GSPE effect is not restricted to a specific photoperiod, our results suggest a more significant effect in the L18 condition.

## 1. Introduction

Apart from Earth’s rotation around its axis, at the same time, the Earth is moving around the Sun creating circannual periods, which define the day length variations and the seasons. Related to this, animals have been adapted to circannual rhythms by a physiological process that depends on an innate long-term timer which is synchronized with the annual environmental cycle (photoperiodic entrainment) [1]. In mammals, this information is received via photoreceptor cells in the retina, which are connected through the retinohypothalamic tract to pineal gland, which converts the photic information through the neurohormone melatonin. Therefore, photoperiodism relies on the way that the pineal gland transduces a photoperiod into a twenty-four-hour melatonin signal, and how its duration is decoded in melatonin-sensitive tissues that control specific aspects of seasonal physiology and behavior [2]. Thus, melatonin codes for day length as it is excreted at night, playing a pivotal role in the control of seasonal and daily changes [3].

In consequence, the study of seasonal adaptations of mammals is important for understanding the underlying regulatory mechanisms implicated, which has great potential for translational research as there is increasing evidence indicating an impact of seasonal timing on several biological processes (e.g., immunity). For example, it has been shown that more than 4000 protein-coding mRNAs in human white blood cells and adipose tissue have seasonal expression profiles, with inverted patterns observed between Europe and Oceania. This shows a seasonal component as part of human immunity and physiology [4]. Furthermore, regarding hepatic lipid metabolism, it has been shown that the fish *Oryzias latipes* showed a greater accumulation of fatty acids in the liver under the short-day condition than under the long-day condition [5]. Additionally, Togo and colleagues demonstrated that the photoperiod regulates feeding and energy metabolism in young growing Fischer 344 rats. These rats preferred a diet with high carbohydrate content compared to one with less carbohydrates under the long-day condition, while no preference for diets was observed under the short-day condition. Moreover, rats under the long-day condition showed an increase in body weight, epididymal fat mass, and plasma leptin levels compared to animals under short-day condition regardless of dietary composition [6]. On the other hand, another season-dependent biological parameter is ROS. In this regard, it has been shown that seasonal changes in the antioxidant defenses enable species to maintain their correct levels of ROS to carry out physiological functions in response to changing physical environmental parameters [7]. Interestingly, Cruz-Carrión and colleagues demonstrated that photoperiods modulate the oxidative stress response to the consumption of local and non-local sweet cherries in rats [8].

This evidence has led, in the last years, to an increasing scientific interest in the study of the influence of circannual rhythmicity in the development of metabolic diseases, and how the addition of bioactive compounds to the diet may help to attenuate these disorders. For this purpose, Fisher 344 rats are known to be a species sensitive to photoperiods, and therefore an excellent model for studying seasonal variation [6].

On the other hand, polyphenols, which are organic compounds found in plants, have become a new field of research in nutrition in recent decades. Among them, proanthocyanidins are powerful naturally derived antioxidants, which have been proven to exhibit anticarcinogenic, anti-inflammatory, antiallergic, antibacterial, antiviral, antidiabetic, immuno-stimulating, neuroprotective, and cardioprotective effects [9,10], as well as to extend the lifespan of animals, apparently by mimicking the beneficial effects of caloric restriction through the increase of SIRT1 expression, which has been recognized as an essential factor for lifespan extension [11,12] In addition, these polyphenolic compounds are able to dampen inflammatory signaling, induce selective apoptosis of senescent cells, and modulate nutrient-sensing pathways. These are biological processes that become dysfunctional with age and are relevant in the pathogenesis of age-related syndrome [13,14]. Interestingly, several in vivo and in vitro experiments demonstrated that supplementation with grape seed procyanidin extract (GSPE) alleviated oxidative stress through the inhibition of lipid peroxidation [15], mitigated endoplasmic reticulum (ER) stress [16], improved mitochondrial function [17], and reduced liver glutathione alteration in obese rat models [18]. Furthermore, GSPE was able to increase normal insulin content and to decrease the number of apoptotic cells in diabetic pancreatic islets [19]. Consequently, these experiments have demonstrated that GSPE is nontoxic, highly bioavailable, and provides significant multiorgan protection. Additionally, GSPE intake in rats has also been shown to produce behavioral changes, such as decreased locomotor activity and decreased food consumption, and modulations on energy expenditure, which could be related to changes observed on mechanisms subjected to seasonal control, such as dopaminergic and somatostatinergic systems [20,21,22]. Consequently, these experiments have demonstrated that polyphenols provide significant multiorgan protection and could modulate seasonal rhythms by acting not only at a central level but also in peripheral tissues, such as liver and adipose tissue, exerting an impact on systemic metabolism. However, little is known about the influence of seasonal variations on the beneficial effects of polyphenols, which may condition their administration schedule. Therefore, the aim of this study was to investigate the hypothetical differential effect of GSPE supplementation on hepatic glucose and lipid metabolism in Fischer 344 rats under different photoperiod conditions.

## 2. Materials and Methods

### 2.1. Grape Seed Proanthocyanidin-Rich Extract

The GSPE was kindly provided by Les Dérivés Résiniques et Terpéniques (Dax, France). According to the manufacturer, the GSPE composition used in this study contained monomers (21.3%), dimers (17.4%), trimers (16.3%), tetramers (13.3%), and oligomers (5–13 units; 31.7%) of proanthocyanidins. The exact phenolic composition of GSPE was determined by HPLC-MS/MS and consisted of catechin (58 μmol/g), dimeric procyanidins (250 μmol/g), epicatechin (52 μmol/g), epigallocatechin (5.50 μmol/g), epicatechin gallate (89 μmol/g), epigallocatechin gallate (1.40 μmol/g), hexameric procyanidins (0.38 μmol/g), pentameric procyanidins (0.73 μmol/g), tetrameric procyanidins (8.8 μmol/g), and trimericprocyanidins (1568 μmol/g) [23].

### 2.2. Experimental Design

A total of 48 12-week-old male Fischer 344 (F344) rats (Charles River Laboratories, Barcelona, Spain) were housed in pairs (in cages) at 22 °C, 55% humidity, and subjected to three different light schedules for 9 weeks to mimic seasonal day lengths: short day photoperiod L6 (*n* = 16, 6 h light and 18 h darkness), normal day or standard photoperiod L12 (*n* = 16, 12 h light and 12 h darkness), and long day photoperiod L18 (*n* = 16, 18 h light and 6 h darkness). Animals were fed with a standard diet (STD) ad libitum. The STD composition was 20% protein, 8% fat, and 72% carbohydrates (Panlab, Barcelona, Spain). Within each photoperiod group, rats were randomly divided into two groups depending on the treatment: 8 STD-fed rats were treated with condensed milk, vehicle (VH), and 8 STD-fed rats were treated with 25 mg/kg GSPE. The treatment lasted four weeks and was orally administered daily using a syringe. During the entire study, rats had free access to water, and body weight and food intake were weekly recorded. After 9 weeks, animals were kept from food for 3 h and then sacrificed by decapitation at 9 am (one hour after light was turned on; ZT1) under anesthesia (sodium pentobarbital, 50 mg/kg per body weight). Blood was collected and serum was obtained by centrifugation (15,000× *g*, 10 min, 4 °C) and stored at −80 °C until analysis. The liver was rapidly weighed, frozen in liquid nitrogen, and stored at −80 °C for further analysis. The Animal Ethics Committee of the Universitat Rovira i Virgili (Tarragona, Spain) approved all procedures (reference number 9495, 18 September 2019) and they were carried out in accordance with Directive 86/609EEC of the Council of the European Union and the procedure established by the Departament d’Agricultura, Ramaderia i Pesca of the Generalitat de Catalunya.

### 2.3. Serum Analysis

Circulating levels of glucose, total cholesterol, HDL and LDL cholesterol and triglycerides (QCA, Barcelona, Spain), were analyzed by colorimetric enzymatic assay kits according to the manufacturer’s instructions.

### 2.4. RNA Extraction

Approximately 20–30 mg of liver tissue was mixed with Trizol^®^ reagent (Thermo Fisher, Madrid, Spain) and homogenized by Tissue Lyser LT (Qiagen, Madrid, Spain). After a 10-min centrifugation (12,000× *g* and 4 °C), the homogenate was placed into a new Eppendorf tube and 120 μL of chloroform was added. Two phases were separated after a 15-min centrifugation (12,000× *g* and 4 °C). The aqueous phase was transferred into a new Eppendorf and 300 μL of isopropanol was added. An overnight incubation (−20 °C) was carried out to extract the microRNA (miRNA). The supernatant was discarded after a 10-min centrifugation (12,000× *g* and 4 °C). The pellet was cleaned twice with 5-min centrifugation (8000× *g* and 4 °C) with 500 μL of ethanol 70%. The supernatant was discarded, and the cleaned pellet was resuspended with 60 μL of nuclease-free water (Thermo Fisher, Madrid, Spain). The RNA concentration (ng/μL) and the purity were measured in Nanodrop ND-1000 spectrophotometer (Thermo Fisher, Madrid, Spain).

### 2.5. Gene Expression Analysis

The cDNA was obtained by a reverse transcription of the RNA extracted using a High-Capacity Complementary DNA Reverse Transcription Kit (Thermo Fisher, Madrid, Spain). The quantitative polymerase chain reactions (qPCRs) were performed in 384-well plates in a 7900HT Fast Real-Time PCR (Thermo Fisher, Madrid, Spain) using iTaq™ Universal SYBR^®^ Green Supermix (Bio-Rad, Barcelona, Spain). The thermal cycle used in all qPCRs was 30 s at 90 °C and 40 cycles of 15 s at 95 °C and 1 min at 60 °C. All liver genes were normalized by the housekeeping gene peptidylprolyl Isomerase A (*Ppia).* The primers used for each gene were obtained from Biomers (Ulm, Germany) (Table 1). The relative expression of each gene was calculated according to the Pfaffl method (2001) and normalized by the control group L12-STD-VH.

### 2.6. Extraction and Measurement of Concentrations of Lipids in Liver

Liver lipids were extracted following the Bligh and Dyer method [24] and levels of hepatic cholesterol, triglycerides, and phospholipids were measured using a colorimetric kit assay (QCA, Barcelona, Spain).

### 2.7. Protein Extraction

A liver tissue portion (20 mg) was mixed with 500 μL of radioimmunoprecipitation assay (RIPA) buffer containing phosphatase inhibitor cocktail 2 and 3, protease inhibitor cocktail, and phenylmethylsulphonyl fluoride. The Eppendorf tube content was homogenized by Tissue Lyser LT (Qiagen, Madrid, Spain). The samples were shaken 30 min at 4 °C and the tube content was placed into a new Eppendorf tube. After a 15-min centrifugation (12,000× *g* and 4 °C), the supernatant was transferred into a new Eppendorf tube. The protein quantification was carried out using a colorimetric bicinchoninic acid (BCA) assay kit (Thermo Fisher, Madrid, Spain) according to the manufacturer instructions.

### 2.8. Determination of Serum Hormones

The analytes melatonin (≥98%), corticosterone (≥98%), 3,3′,5-triiodo-L-thyronine (T3) (≥95%), L-thyroxine (T4) (≥98%) were obtained from Sigma-Aldrich (St. Louis, MO, USA). The internal standards L-thyroxine-D4 (≥98%) and 3,3′,5-triiodo-L-thyronine (≥98%) were purchased from Sigma-Aldrich (St. Louis, MO, USA); melatonin-D4 (≥98%)) was purchased from Cluzeau Info Labo (Sainte-Foy-la-Grande, France); and corticosterone-D8 (≥98%) was obtained from NeoChema (Bodenheim, Germany). Formic acid and ethyl acetate were obtained from Sigma-Aldrich (Madrid, Spain) and methanol from MERK (Madrid, Spain). All solvents and reagents used in the present study were HPLC grade. HPLC grade water was obtained by ultrafiltration (Millipore Milli Q system, Bedford, MA, USA). The standard stock (1 mg/mL) and internal standard (100 μg/mL) solutions were prepared in methanol. The working and calibration solutions were prepared in a water–methanol solution (1:1, *v*/*v*).

For determination of melatonin, corticosterone, triiodothyronine (T3), and thyroxine (T4) levels, serum samples were thawed at 4 °C. Then, 50 μL of serum was mixed with 250 μL of methanol containing the internal standard (2 ng/mL). Then, the mixture was vortexed and centrifuged for 5 min at 4 °C and 15,000 rpm. The supernatant was transferred to a new tube and mixed with 700 μL of 0.1% formic acid in water. The sample was loaded to an SPE tube previously conditioned with methanol and 0.1% formic acid in water. The cartridge was washed with 0.1% formic acid in water and dried under high vacuum. The compounds were eluted with 500 μL of methanol. Samples were evaporated in a SpeedVac at 45 °C and reconstituted with 50 μL of water:methanol (60:40, *v*/*v*) and transferred to a glass vial for analysis. Simultaneous detection and quantification of hormones levels were achieved using liquid chromatography coupled to triple quadrupole mass spectrometry (LC-QqQ).

### 2.9. Metabolomic Analysis

Metabolomic analysis of the 48 rat liver samples was performed at the Centre for Omic Sciences (COS, Tarragona, Spain) using gas chromatography coupled with quadrupole time-of-flight mass spectrometry (GC-qTOF model 7200, Agilent, Santa Clara, CA, USA). The extraction was performed by adding 400 µL of methanol:water (8:2)-containing internal standard mixture to liver samples (approximately 10–20 mg). Then, the samples were mixed and homogenized on a bullet blender using a stainless-steel ball, incubated at 4 °C for 10 min, and centrifuged at 19,000× *g*; supernatant was evaporated to dryness before compound derivatization (methoximation and silylation). The derivatized compounds were analyzed by GC-qTOF. Chromatographic separation was based on the Fiehn Method [25] using a J&W Scientific HP5-MS film capillary column (30 m × 0.25 mm × 0.25 µm, Agilent, Santa Clara, CA, USA) and helium as carrier gas with an oven program from 60 to 325 °C. Ionization was done by electronic impact (EI), with electron energy of 70 eV and operating in full-scan mode. Identification of metabolites was performed using commercial standards and by matching their EI mass spectrum and retention time to a metabolomic Fiehn library (from Agilent, Santa Clara, CA, USA), which contains more than 1400 metabolites. After putative identification of metabolites, they were semi-quantified in terms of internal standard response ratio.

### 2.10. Statistical Analysis

Data are reported as mean ± standard error of the mean (SEM). Serum and liver biochemical profile, liver weight, glucose-related hepatic metabolites, and liver gene expression were subjected to Student’s *t* test and one- and two-way analysis of variance (ANOVA) with the least significant difference test (LDS) for post hoc comparisons using the computer program SPSS version 25 (SPSS Inc., Chicago, IL, USA). Graphics were done by GraphPad Prism 8 software (San Diego, CA, USA). For all analyses, a probability (*p*) value of <0.05 was considered statistically significant.

## 3. Results

### 3.1. Photoperiod Strongly Influences Body Weight Gain of F344 Rats

**Figure 1:**
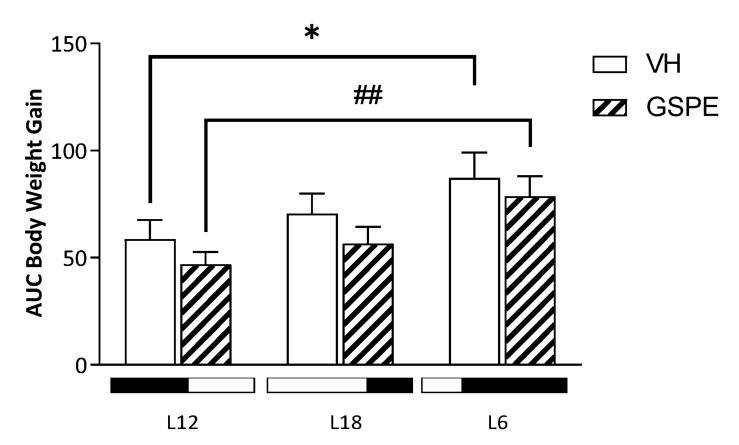
Body weight gain as area under the curve (AUC) of Fischer 344 rats treated with VH or GSPE exposed to standard (12 h light:12 h dark), long (18 h light:6 h dark), or short (6 h light:18 h dark) photoperiods fed with STD diet (*n* = 8). The results are presented as the mean ± SEM. * indicates significant differences within VH groups (Student’s *t* test, * *p* < 0.05); # indicates significant differences within GSPE groups (Student’s *t* test, ## *p* < 0.01).

As shown in Figure 1, irrespective of treatment, rats exposed to a short photoperiod showed significantly higher body weight gain than animals subjected to the standard photoperiod (L12-VH vs. L6-VH; *p* = 0.045) (L12-GSPE vs. L6-GSPE; *p* = 0.0049). No differences were observed between the L12 and L18 groups or between L18 and L6 in relation to body weight gain. Regarding food intake, we observed no differences among the groups (Supplementary Figure S1).

### 3.2. The Effect of GSPE on Serum Parameters Depends on Photoperiod

As shown in Table 2, serum triglycerides levels of L12-GSPE animals tend to decrease compared to L12-VH (*p* = 0.07), whereas an increase in L6-GSPE animals compared to L12-GSPE group is observed (*p* = 0.01). Serum glucose values are elevated in L18-GSPE animals compared to L12 counterparts (*p* = 0.001), showing clear interaction between treatment and photoperiod. In addition, L6 photoperiod showed even higher values of serum glucose when compared to other VH groups, L12 vs. L6 (*p* = 0.004) and L18 vs. L6 (*p* = 0.001). Interestingly, L6-GSPE rats showed the highest serum glucose levels, raising its value around 15 percent compared to its VH, and showed a significant increase of total serum cholesterol compared to its control (*p* = 0.006). Additionally, a photoperiod effect is seen in HDL levels as animals exposed to short photoperiod exhibit lower values; on the other hand, LDL levels are affected not only by photoperiod but also by treatment showing a decrease in LDL values only on L18-GSPE rats.

### 3.3. The Exposure to Different Photoperiods Altered the Expression of Circadian Rhythm-Related Genes in the Liver of GSPE-Treated Rats

**Figure 2:**
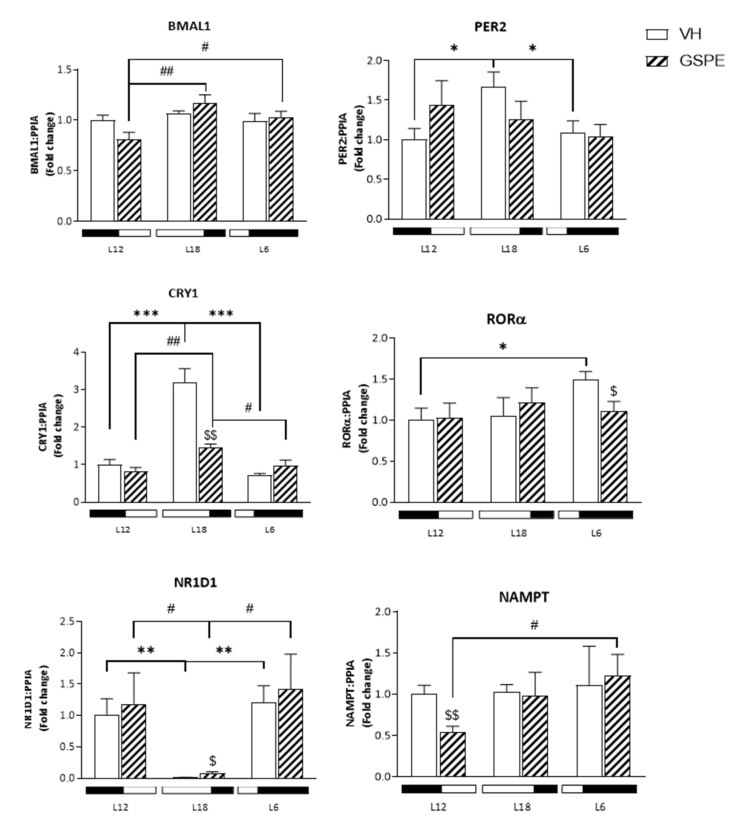
mRNA expression levels of clock genes in the liver of Fischer 344 rats treated with VH or GSPE exposed to standard (12 h light:12 h dark), long (18 h light:6 h dark), or short (6 h light:18 h dark) photoperiods fed with STD diet (*n* = 8). The results are presented as the mean ± SEM. * indicates significant differences within VH groups (Student’s *t* test, * *p* < 0.05, ** *p* < 0.01, *** *p* < 0.001); # indicates significant differences within GSPE groups (Student’s *t* test, # *p* < 0.05, ## *p* < 0.01); $ indicates significant differences between treatments (VH vs. GSPE) (Student’s *t* test, $ *p* < 0.05, $$ *p* < 0.01).

We analyzed the hepatic mRNA expression of key clock genes to try to find correlations with the changes in serum parameters under different photoperiods in response to GSPE treatment. The gene expression of the brain and muscle Arnt-like protein-1 (*Bmal1*) gene was higher in L18 and L6-GSPE rats compered to L12-treated ones (*p* = 0.006 and 0.04, respectively), whereas expression levels of Period circadian clock 2 (*Per2*) only showed changes in VH groups with an increase in L18 compared to L12 and L6 photoperiods (*p* = 0.02 and 0.03, respectively) (Figure 2). Additionally, GSPE lowers mRNA relative levels of hepatic Cryptochrome circadian clock 1 (*Cry1*) gene in L18 (*p* = 0.002), which is highly upregulated in L18-VH. The *Bmal1* activator RAR-related orphan receptor alpha (*Rorα)* gene expression is lower in L6-GSPE compared to L6-VH animals (*p* = 0.048), while L6-VH rats exhibit a higher expression level than L12-VH (*p* = 0.03). Expression of nuclear receptor subfamily 1 group D member 1 gene (*Nr1d1*), a *Bmal1* repressor, is modulated by light showing a totally repressed expression in animals exposed to a long photoperiod while GSPE treatment slightly increased its expression (*p* = 0.04). Regarding Nicotinamide phosphoribosyltransferase (*Nampt*) gene expression, it decreased with GSPE treatment in L12 (*p* = 0.008) and increased when compared with L6-GSPE rats (*p* = 0.046).

### 3.4. Melatonin and Hormones from the Hypothalamus Pituitary Adrenal (HPA) Axes Have a Seasonal Variation which Is Affected by GSPE Consumption

**Figure 3:**
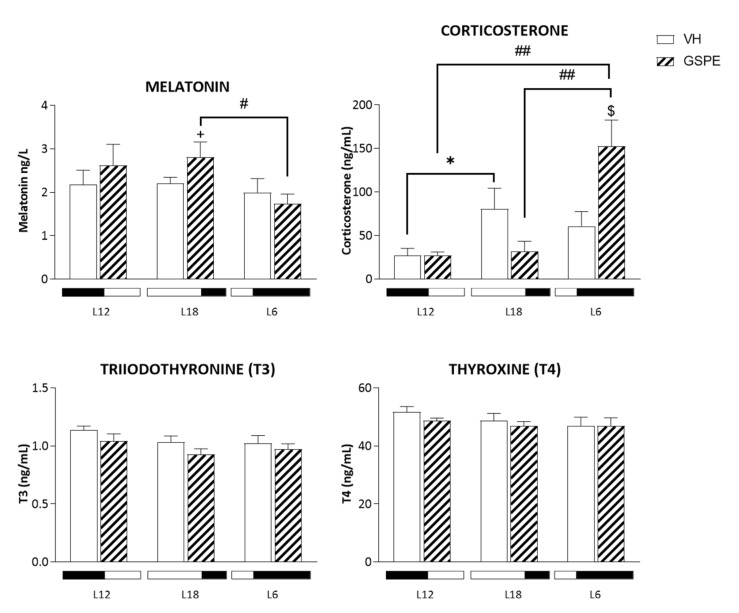
Concentration of serum hormones in response to different photoperiod exposure and GSPE treatment in animals fed a STD for 9 weeks. Fischer 344 rats fed a STD diet were treated with VH or GSPE and exposed to standard (12 h light:12 h dark), long (18 h light:6 h dark), or short (6 h light:18 h dark) photoperiods (*n* = 8). The results are presented as the mean ± SEM. * indicates significant differences within VH groups (Student’s *t* test, * *p* < 0.05); # indicates significant differences within GSPE groups (Student’s *t* test, # *p* < 0.05, ## *p* < 0.01); $ indicates significant differences between treatments (VH vs. GSPE) (Student’s *t* test, $ *p* < 0.05). + indicates tendency between VH and GSPE treatment in L18 using Student’s *t* test (+ *p* = 0.050).

Serum levels of corticosterone, T3 and T4, together with melatonin were analyzed. In this sense, Figure 3 shows significant increase of corticosterone levels in L6-GSPE animals (*p* = 0.01 vs. L6-VH, *p* = 0.004 vs. L12-GSPE, and *p* = 0.002 vs. L18-GSPE), whereas the L18 non-treated group shows an increase compared to the standard photoperiod (*p* = 0.05). In the case of melatonin, GSPE raised the levels of this hormone in the L18 photoperiod (*p* = 0.05); meanwhile, non-significant differences were found in T3 and T4 levels.

### 3.5. Lipid Liver Profile

A clear photoperiod effect is seen in the levels of liver triglycerides, with an increase in L6 compared to standard photoperiod (*p* = 0.007). In addition, GSPE rats subjected to short photoperiod present higher values than L18 (*p* = 0.02) and L12 GSPE animals (*p* = 0.001) (Table 3). Total cholesterol, phospholipids, and liver weight showed no significant differences among groups.

### 3.6. mRNA Levels of Key Hepatic Lipid and Glucose-Metabolic Regulators Varies due to Photoperiod and GSPE Treatment

**Figure 4:**
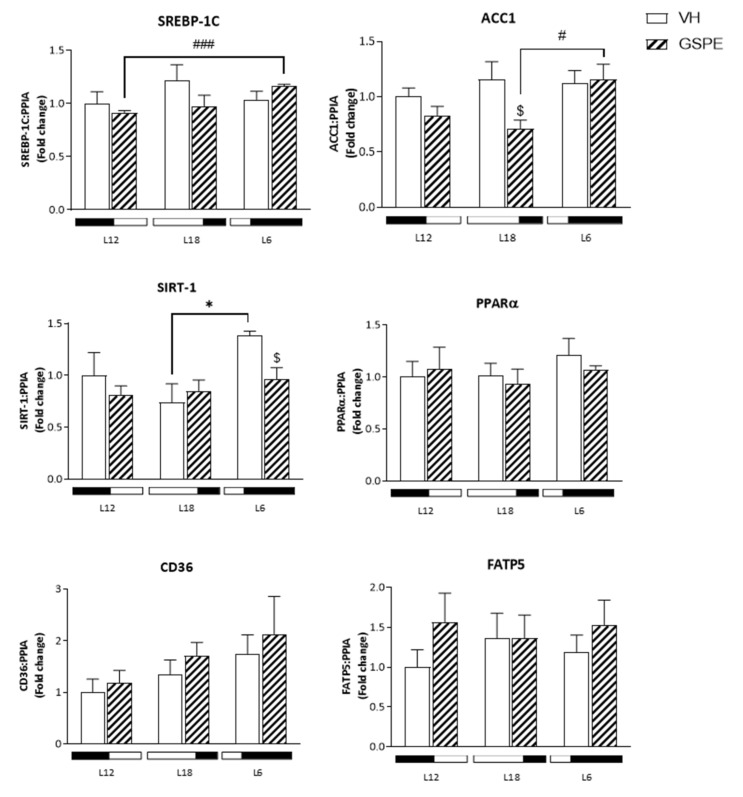

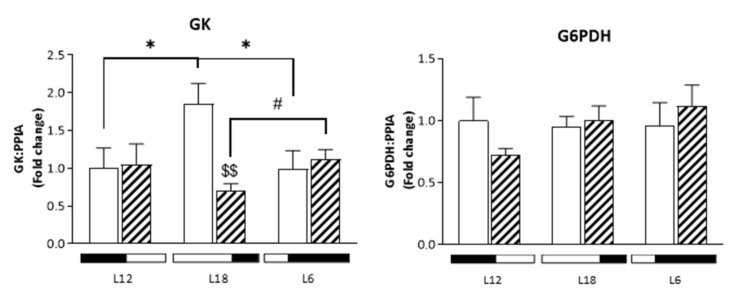
mRNA expression levels of liver lipid and glucose metabolism of Fischer 344 rats treated with VH or GSPE exposed to standard (12 h light:12 h dark), long (18 h light:6 h dark) or short (6 h light:18 h dark) photoperiods fed with STD diet (*n* = 8). The results are presented as the mean ± SEM. * indicates significant differences within VH groups (Student’s *t* test, * *p* < 0.05); # indicates significant differences within GSPE groups (Student’s *t* test, # *p* < 0.05, ### *p* < 0.001); $ indicates significant differences between treatments (VH vs. GSPE) (Student’s *t* test, $ *p* < 0.05, $$ *p* < 0.01).

In order to assess the effect of GSPE treatment on liver lipid and glucose metabolism. mRNA levels of genes implicated in both processes were analyzed. As shown in Figure 4. mRNA levels of SREBP-1c in L6-GSPE rats are higher compared to L12 ones (*p* = 0.001). Moreover. *Acacα* mRNA levels are also increased in L6-GSPE animals (*p* = 0.03). whereas GSPE treatment decreases the expression of this gene in a long photoperiod (*p* = 0.04). Sirtuin 1 (*Sirt1*) gene expression is higher in L6-VH compared to L18-VH animals showing a clear photoperiod effect (*p* = 0.024). Curiously. this expression is decreased when treated with GSPE in L6 (*p* = 0.023). Glucokinase (*Gk*) showed an 85 percent increase in its expression in L18-VH animals compared to other photoperiods (*p* = 0.019 vs. L12-VH and *p* = 0.017 vs. L6-VH). whereas GSPE treatment decreased by 62 percent *Gk* mRNA levels in L18 (*p* = 0.004). Other metabolic genes were analyzed such as *Pparα. Cd36. Fatp5*. and *G6pdh.* although no significant differences were found among groups.

### 3.7. Analysis of Glucose-Related Metabolites Reveals Photoperiod Influence over GSPE Effect

**Figure 5:**
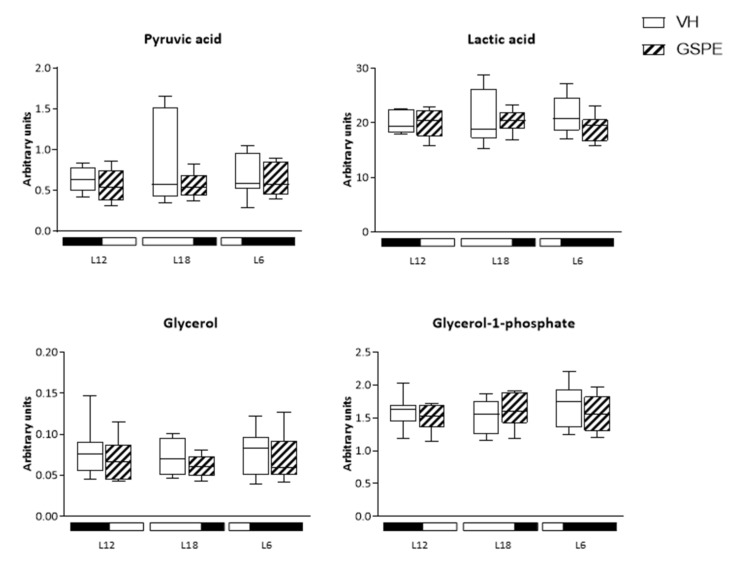

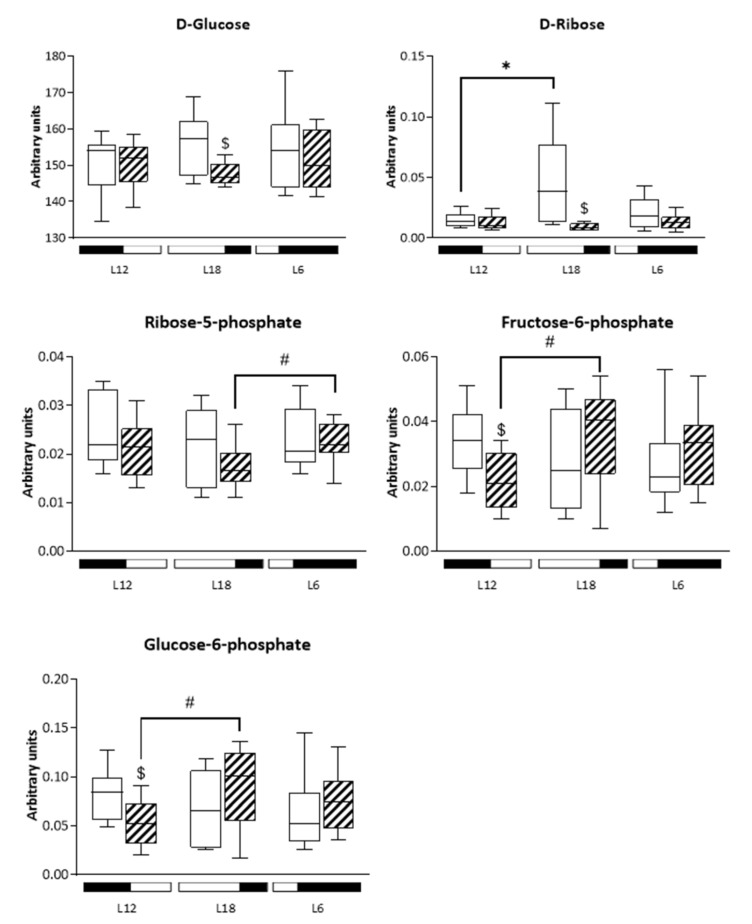
Metabolites related to hepatic glucose metabolism. Metabolites of glucose metabolism in the liver of Fischer 344 rats treated with VH or GSPE exposed to standard (12 h light:12 h dark), long (18 h light:6 h dark), or short (6 h light:18 h dark) photoperiods fed with STD diet (*n* = 8). The results are presented as the mean ± SEM. * indicates significant differences within VH groups (Student’s *t* test, * *p* < 0.05); # indicates significant differences within GSPE groups (Student’s *t* test, # *p* < 0.05); $ indicates significant differences between treatments (VH vs. GSPE) (Student’s *t* test, $ *p* < 0.05).

Liver metabolites related to glucose pathways were studied and plotted in boxplots to visualize their concentrations in and between photoperiods and GSPE treatment. As it is seen in Figure 5, levels of D-glucose are lower in L18-GSPE animals compared to its VH (*p* = 0.027), as well as for D-ribose which shows a decrease in L18-GSPE animals but is increased in L18-VH when compared to L12-VH (*p* = 0.043). In the case of ribose-5-phosphate, L18-GSPE rats show lower levels than L6-GSPE animals (*p* = 0.034). Glucose-6-phosphate and fructose-6-phosphate show a similar pattern, with higher concentration levels in non-treated L12 animals (VH vs. GSPE) (*p* = 0.033 and 0.018, respectively). When compared between GSPE groups, L12 rats exhibit lower levels of these metabolites than in L18 animals (*p* = 0.043 in the case of glucose-6-phosphate and *p* = 0.028 in fructose-6-phosphate), whereas there is a tendency of increasing levels of fructose-6-phosphate in L6-GSPE rats compared to L12 counterparts (*p* = 0.055) (Supplementary Tables S1 and S2).

### 3.8. Photoperiod Affects the GSPE Effect on ER Stress Genes

We measured the relative gene expression of the following key ER stress genes: *Atf4*. *Atf6*. *Grp78*. *s-Xbp1*. and *Chop*.

**Figure 6:**
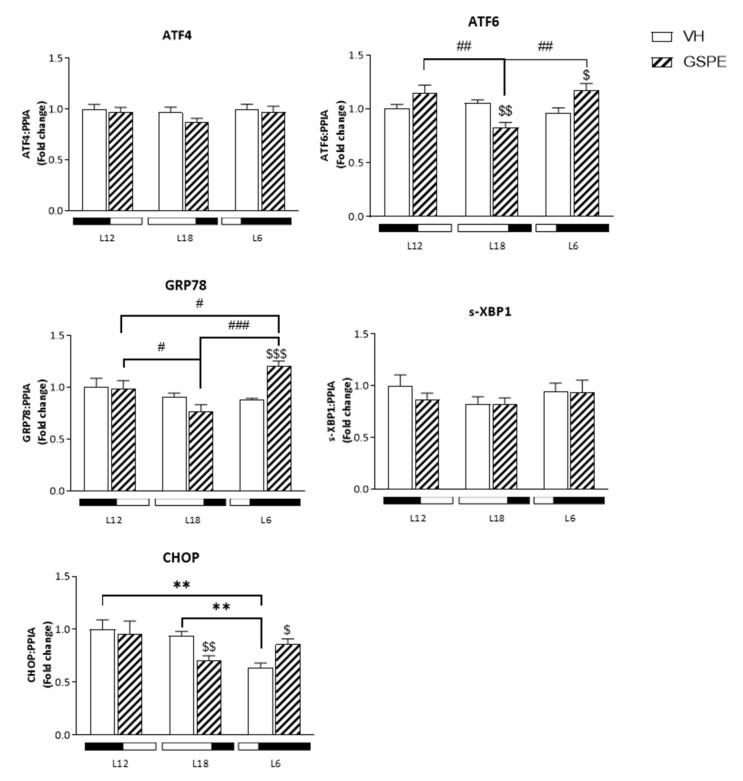
mRNA expression levels of ER stress genes of Fischer 344 rats treated with VH or GSPE exposed to standard (12 h light:12 h dark), long (18 h light:6 h dark), or short (6 h light:18 h dark) photoperiods fed with STD diet (*n* = 8). The results are presented as the mean ± S.E.M. * indicates significant differences within VH groups (Student’s *t* test, ** *p* < 0.01); # indicates significant differences within GSPE groups (Student’s *t* test, # *p* < 0.05, ## *p* < 0.01, ### *p* < 0.001); $ indicates significant differences between treatments (VH vs. GSPE) (Student’s *t* test, $ *p* < 0.05, $$ *p* < 0.01, $$$ *p* < 0.001).

As shown in Figure 6. the relative gene expression of *Grp78* in L6 GSPE animals is the highest of all treatments (*p* = 0.001 vs. L6-VH and vs. L18-GSPE. *p* = 0.037 vs. L12-GSPE). whereas L18 GSPE-treated animals showed the lowest expression of this gene. Similar results are seen in the *Atf6* gene expression for L18 GSPE group (*p* = 0.002 vs. L18-VH. *p* = 0.005 vs. L12-GSPE. *p* = 0.001 vs. L6-GSPE). and it is also seen an increase in the expression of this gene in L6 GSPE animals compared to its control group (*p* = 0.03). In the same line. *Chop* gene expression is decreased in L18 GSPE-treated animals (*p* = 0.005 vs. L18-VH) and increased in L6 GSPE group (*p* = 0.013 vs. L6-VH).

## 4. Discussion

Intrinsically synchronized with the natural year, circannual rhythms are responsible for scheduling seasonal activities in relation to the outer environmental cues. These rhythms function as a sensor to adjust the physiology and behavior of an organism to the periodically changing conditions [26].

Polyphenols have been widely described as compounds able to regulate metabolic syndrome-associated disorders which can also interact with the biological rhythms by affecting the expression of clock genes [27]. Consequently, the season of the year in which polyphenols are consumed could likely modify their ability to restore metabolic disorders. The current study aims to investigate whether these adaptative mechanisms can also modulate the effect on liver metabolism of a specific polyphenol extract.

The first conclusion that we can draw from our results is the slight alteration on metabolic health due to short photoperiod exposure (L6) in rats (discussed below). This result strongly correlates with previous findings in which L6 rats exhibited markedly altered glucose homeostasis and fatty acid uptake and oxidation when compared to animals exposed to longer photoperiods [28]. More precisely, depending on the light schedules, we observed variations in hepatic triglycerides levels, some serum parameters (i.e., glucose and triglycerides), hormones (corticosterone and melatonin), and hepatic glucose metabolites, which could be linked to changes in the expression of several hepatic circadian clock genes such as *Bmal1*, *Cry1*, and *Nr1d1*, as well as lipogenic (SREBP-1c, *Acacα*) and ER stress (*Atf6*, *Grp78*, and *Chop*) genes.

Numerous studies have documented that the exposure to different day length schedules has an impact on physiology, behavior, and reproduction in animals that are exposed to different photoperiods. In fact, many of these studies have been carried out using photoperiod-sensitive F344 rats. In the present study, we observed no changes in food intake between the different photoperiods, which was in agreement with previous studies using F344 rats subjected to STD diet [28]. On the other hand, we observed differences in body weight gain which was increased in rats exposed to the short photoperiod compared to the standard, and this effect was independent of the treatment. This could be attributed to the hours of sleep L6 animals had, as it has been reported that short sleep duration could be an independent risk factor for weight gain [29]. These results, however, clearly differed from other studies using the F344 strain, in which the exposure to a short photoperiod reduced body mass compared to long-day photoperiods. These could be explained by the differences in age and reproductive status of the animals used and the duration of the experiments [30,31,32] Nevertheless, human studies have shown that lower levels of melatonin secretion in the autumn-winter period can increase appetite and lead to weight gain [33]. These results are in agreement with our present results, regarding the lower melatonin levels in the L6 photoperiod and the weight gain.

Regarding the effect of GSPE administration, our results clearly showed a differential GSPE modulation on the hepatic glucose and lipid metabolism in a seasonal-dependent manner.

The liver, the main organ involved in cholesterol homeostasis, synthesizes very low-density lipoproteins (VLDL) to transport triacylglycerol from the hepatocyte to peripheral tissues. A remnant is formed after this particle is metabolized in extrahepatic tissues, and part of this remnant is converted into LDL. It is known that one of the most important risk factors of atherosclerotic disease is the concentration of cholesterol carried in LDL [34], both parameters also being closely related to the prevalence of non-alcoholic fatty liver disease [35]. Humans show total cholesterol and LDL levels higher in winter than in summer [36,37], partly due to seasonal variations in food intake and physical activity which lead to an increase of body weight in winter [38]. Accordingly with these observations in humans, in our study, rats exposed to the short photoperiod (L6) showed the highest levels of serum cholesterol, which GSPE was unable to reduce, contrary to the anticholesterolemic activity showed by GSPE on rats exposed to a long photoperiod (L18). Nevertheless, we have to keep in mind that such cholesterol levels are not associated, in this case, with any pathological state.

Our results also showed different responses regarding the maintenance of glucose homeostasis between the experimental groups. In high glycemic conditions, glucose is phosphorylated to glucose-6-phosphate by GK in the hepatocyte. Glucose-6 phosphate serves as a metabolic link connecting glycolysis with the pentose phosphate pathway, de novo lipogenesis, and glycogen synthesis by inducing glycogen synthase activation in hepatocytes [39,40,41]. These findings agree with our results in which L18-GSPE animals display the highest levels of glucose-6-phosphate, consistent with the elevated blood glucose levels showed in this group, meanwhile L6-GSPE animals did not show this increase in glucose-6-phosphate concentrations despite their elevated bloodstream glucose.

In addition, it is important to note that in conditions of ribose 5-phosphate requirements, the cells transform glucose 6-phosphate into fructose 6-phosphate and glyceraldehyde 3-phosphate through the glycolytic pathway. These molecules are then converted into ribose 5-phosphate via the reverse steps of the nonoxidative phase. When NADPH is needed, the cell firstly undergoes oxidative reactions followed by non-oxidative reactions and finally gluconeogenesis. As a result, ribose 5-phosphate is recycled back into glucose 6-phosphate, which is used to synthesize NADPH molecules. A cell that needs ATP and NADPH undergoes both oxidative and non-oxidative phases, thereby synthesizing NADPH and transforming the ribose 5-phosphate into glycolytic intermediates that can be used to synthesize ATP [42,43]. Our results showed levels of fructose-6-phosphate also higher in L18-GSPE animals whereas L6-GSPE rats showed elevated levels of ribose-5-phosphate. Therefore, based on these results, it is possible to suggest that L18-GSPE rats have enhanced the glycolytic metabolic pathway, whereas L6-GSPE animals display stimulated gluconeogenesis. This observation in L6-GSPE rats may be partly explained by the variation of corticosterone circulating levels between the experimental groups. It is known that glucocorticoids increase fat storage and glucose production in the liver [44,45] and L6-GSPE rats showed corticosterone levels five times higher than the others GSPE groups.

We also analyzed the levels of melatonin based on its activity as scavenger of free radicals, thus increasing the activity of antioxidant enzymes (i.e., glutathione peroxidase, superoxide dismutase and catalase), which are crucial for maintaining liver function and determining the protective role of melatonin on liver damage [46,47,48,49,50,51]. We observed differences in melatonin levels between L18 and L6 that can be attributed to the different hours of light exposure. As melatonin is released during the dark phase, animals exposed to long photoperiod have fewer hours of darkness than animals exposed to short photoperiod. As they were sacrificed at the same time, the peak of melatonin will be more pronounced in L18 than in L6 rats because the dark phase of L18 animals is closer to the sacrifice time point, meanwhile in short photoperiods, melatonin is released 12 h earlier, at the beginning of their dark phase. In addition to light exposure, it is known that GSPE can modulate melatonin levels in plasma [52]. In accordance with other parameters that seem to confer beneficial properties to the L18-GSPE group, our results showed a more evident increase in melatonin levels in such animals.

Past research has demonstrated an association between ER stress and hepatic lipogenesis, as ER plays an important role not only in protein folding but also in lipid synthesis and metabolism [53]. Interestingly, prior studies have shown that ER stress can activate SREBPs, resulting in the upregulation of lipogenic genes such as *Fasn* and *Acacα*, as well as of transcription factors and proteins involved in hepatic cholesterol and in triglyceride synthesis [54,55]. Moreover, another study demonstrated that activation of SREBP-1 by ER stress induces hepatic triglyceride accumulation in mice [56]. In addition, it was found that culture liver cells with saturated fatty acids or with triglyceride-rich particles disrupt ER homeostasis and activates the expression of ER stress genes and proteins [57,58]. These findings are consistent with those obtained in our study as a downregulation in the expression of *Atf6* and *Chop,* genes involved in ER stress, in L18-GSPE rats, has been observed together with the decrease in the expression of lipogenic genes and liver triglycerides. In contrast, L6-GSPE rats, those with a more adverse lipid profile, overexpressed *Grp78, Atf6*, and *Chop*. Therefore, ER stress activation may play an early and critical role in the cellular response to fatty acid overload. In concordance with these results, levels of *Sirt1* were found to be downregulated in L6-GSPE animals. It is known that *Sirt1* is essential for the regulation of liver lipid metabolism and gluconeogenesis [59]. In more detail, it has been shown that *Sirt1* negatively regulates the expression of genes involved in glycolysis, triglyceride synthesis, and lipid metabolism [60]. This result strongly agrees with another study which demonstrated that hepatic lipid concentrations negatively correlated with *Sirt1* mRNA levels in animals supplemented with GSPE [61]. In addition, it has been demonstrated that SREBP-1c is necessary for *Gk* expression in hepatocytes [62], as well as the requirement of GK for the expression of lipogenic genes such as *Fasn* and *Acacα* [63]. Thus, glucose phosphorylation by hepatic GK is not only a crucial event for glucose metabolism but also for lipid metabolism in the liver. Accordingly, in our results, the decrease of lipogenic genes expression in L18-GSPE rats was also accompanied by a dramatic reduction in *Gk* liver expression. In this regard, a summary table of the main metabolic changes due to the GSPE treatment in each photoperiod has been added in Supplementary Table S3.

Finally, one of the duties of the mammalian circadian clock is to measure photoperiodic time, acting as an accurate natural predictor of annual phases, making it possible to adapt to seasonal changes [64]. We detected changes at both photoperiod and treatment comparisons, as differences between treatments were observed in L12 (*Nampt*) as well as in L18 (*Cry1, Nr1d1*) and in L6 (*Rorα).* According to these results, it is likely that a circadian rhythm mismatch in L18-VH animals and GSPE treatment is able to recover from this disturbance in these animals exposed to a long photoperiod. It has been shown that clock-core genes contribute to the regulation of glucose and lipid metabolism in the liver [65]. In this regard, it is plausible to suggest that regulation seen on L18-GSPE animals on lipogenic genes may have a relationship with the restoration of circadian mismatch seen on animals exposed to L18 photoperiod. However, one limitation is that we were able to measure the expression of circadian rhythm-related genes only at a single time point (ZT 1). Therefore, an analysis conducted over a 24-h period at different daily time points would be necessary to confirm this hypothesis. Nevertheless, our results correlate with previous studies showing that the exposure to short and long photoperiods caused a disruption in the expression of hepatic circadian clock impairing lipid and glucose metabolism in the liver [66]. Furthermore, in order to depict the global metabolic scenario regarding our experimental approach, it will be also important to carry out proteomics and epigenomics approaches to investigate further into the physiological outcomes of photoperiods and treatments.

## 5. Conclusions

The present study highlights the importance of circannual rhythms in regulating metabolic homeostasis and suggests that seasonal variations (long or short photoperiods) affect hepatic metabolism. Furthermore, our findings suggest that GSPE effects vary among photoperiods and could improve the consequences related to a change in photoperiod (e.g., partial disruption in the circadian rhythmicity of clock genes, slight alterations on lipid and glucose metabolism) which could be associated with obesity promotion. Finally, our results suggest that the GSPE effect, although not restricted to any specific photoperiod, is especially relevant in the L18 photoperiod under physiological conditions.

## Figures and Tables

**Table 1:** Nucleotide sequences of primers used for real-time quantitative PCR.

Gene	Forward Primer (5′ to 3′)	Reverse Primer (5′ to 3′)
*Acacα*	TGCAGGTATCCCCACTCTTC	TTCTGATTCCCTTCCCTCCT
*Atf4*	TATGGATGGGTTGGTCAGTG	CTCATCTGGCATGGTTTCC
*Atf6*	GACTGGGAGTCCACGTTGTT	GAACAGGAGTCTGTGGACCG
*Bmal1*	GTAGATCAGAGGGCGACGGCTA	CTTGTCTGTAAAACTTGCCTGTGAC
*Cd36*	GTCCTGGCTGTGTTTGGA	GCTCAAAGATGGCTCCATTG
*Chop*	AAGATGAGCGGGTGGCAGCG	CCGGTTTCTGCTTTCAGGTGTGGT
*Cry1*	TGGAAGGTATGCGTGTCCTC	TCCAGGAGAACCTCCTCACG
*Fatp5*	CCTGCCAAGCTTCGTGCTAAT	GCTCATGTGATAGGATGGCTGG
*G6pdh*	ACCAGGCATTCAAAACGCAT	CAGTCTCAGGGAAGTGTGGT
*Gk*	CTGTGAAAGCGTGTCCACTC	GCCCTCCTCTGATTCGATGA
*Grp78*	CTACGAAGGTGAACGACCCC	ATTTCTTCAGGGGTCAGGCG
*Nampt*	CTCTTCACAAGAGACTGCCG	TTCATGGTCTTTCCCCCACG
*Nr1d1*	ACAGCTGACACCACCCAGATC	CATGGGCATAGGTGAAGATTTCT
*Per2*	CGGACCTGGCTTCAGTTCAT	AGGATCCAAGAACGGCACAG
*Pparα*	CGGCGTTGAAAACAAGGAGG	TTGGGTTCCATGATGTCGCA
*Ppia*	CCAAACACAAATGGTTCCCAGT	ATTCCTGGACCCAAAACGCT
*Rorα*	CCCGATGTCTTCAAATCCTTAGG	TCAGTCAGATGCATAGAACACAAACTC
*Srebp1c*	CCCACCCCCTTACACACC	GCCTGCGGTCTTCATTGT
*s-Xbp1*	AAACAGAGTAGCAGCACAGACTGC	TCCTTCTGGGTAGACCTCTGGGAG

**Table 2:** Concentration of serum parameters in response to different photoperiod exposure and GSPE treatment in animals fed a STD diet for 9 weeks.

Serum Parameters	L12		L18		L6		2wA
	STD-VH	STD-GSPE	STD-VH	STD-GSPE	STD-VH	STD-GSPE	
Glucose (mg/dL)	110.96 ± 4.67 ^a^	102.72 ± 2.63 ^a^	110.34 ± 2.86 ^a^	141.81 ± 3.06 ^b^	136.34 ± 5.82 ^b^	154.49 ± 6.65 ^c^	T, P, T*P
Cholesterol (mg/dL)	98.53 ± 5.30 ^a^	104.54 ± 6.98 ^ab^	107.15 ± 7.06 ^ab^	108.36 ± 4.84 ^ab^	98.60 ± 3.61 ^a^	117.47 ± 4.15 ^b^	T
Triglycerides (mg/dL)	56.13 ± 4.08 ^ab^	46.73 ± 2.02 ^b^	58.72 ± 4.69 ^ab^	60.71 ± 5.84 ^ab,*^	52.90 ± 3.25 ^ab^	64.73 ± 6.93 ^a^	
HDL (mg/dL)	35.33 ± 1.43 ^a^	32.11 ± 2.28 ^ab^	28.57 ± 2.25 ^bc^	31.38 ± 1.13 ^abd^	25.44 ± 1.27 ^c^	26.93 ± 2.49 ^cd^	P
LDL (mg/dL)	30.55 ± 3.20 ^a^	34.79 ± 2.59 ^a^	32.47 ± 3.33 ^a^	20.80 ± 1.94 ^b^	19.45 ± 1.47 ^b^	19.43 ± 2.08 ^b^	P, T*P

Serum parameters of Fischer 344 rats fed a STD diet and exposed to three different photoperiods for 9 weeks, supplemented with vehicle or GSPE for the last 4 weeks. Data are given as mean ± SEM. (*n* = 8). One- and two-way ANOVA following by LSD post hoc tests were performed to compare the values between groups and significant differences (*p* ≤ 0.05) were represented with different letters (a, b, c, d). * Indicates tendency between L12-STD-GSPE and L18-STD-GSPE groups (*p* = 0.052). P, photoperiod effect. T, treatment effect. T*P, interaction between photoperiod and treatment. Abbreviations: HDL, high-density lipoprotein; LDL, low-density lipoprotein.

**Table 3:** Liver lipids parameters in response to different photoperiod exposure and GSPE treatment in animals fed a STD for 9 weeks.

Parameters	L12		L18		L6		2wA
	STD-VH	STD-GSPE	STD-VH	STD-GSPE	STD-VH	STD-GSPE	
Cholesterol (mg/g)	1.19 ± 0.06 ^ab^	1.27 ± 0.08 ^a^	1.04 ± 0.07 ^b^	1.09 ± 0.07 ^ab^	1.14 ± 0.05 ^ab^	1.12 ± 0.06 ^ab^	
Triglycerides (mg/g)	2.62 ± 0.17 ^b^	2.98 ± 0.10 ^ab^	3.44 ± 0.35 ^abc^	3.34 ± 0.13 ^a^	3.68 ± 0.29 ^ac^	3.84 ± 0.15 ^c^	P
Phospholipids (mg/g)	6.82 ± 0.27 ^a^	6.86 ± 0.36 ^a^	6.3 ± 0.34 ^a^	6.32 ± 0.26 ^a^	6.92 ± 0.34 ^a^	7.05 ± 0.42 a	
Liver weight (g)	14.78 ± 0.5 ^a^	15.25 ± 0.6 ^a^	15.21 ± 0.7 ^a^	15.13 ± 0.61 ^a^	15.6 ± 0.47 ^a^	14.68 ± 0.61 ^a^	

Lipid parameters in the liver of Fischer 344 rats fed a STD diet and exposed to three different photoperiods for 9 weeks, supplemented with vehicle or GSPE for the last 4 weeks. Data are given as mean ± SEM. (*n* = 8). One- and two-way ANOVA following by LSD post hoc tests were performed to compare the values between groups and significant differences (*p* ≤ 0.05) were represented with different letters (a, b, c). P, photoperiod effect.

## Data Availability

The data presented in this study are available on request from the corresponding author. The data are not publicly available due to lack of platform to publish them.

## Supplementary Materials

The following are available online at https://www.mdpi.com/article/10.3390/biom12060839/s1, Table S1. Treatment comparison between metabolites related to hepatic glucose metabolism; Table S2. Photoperiod comparison between metabolites related to hepatic glucose metabolism; Table S3. Summary of the main metabolic changes due to the GSPE treatment in each photoperiod compared to its respective VH control; Figure S1. Average of weekly food intake (kJ) of different photoperiods.

## References

[1] Wood S., Loudon A., Wood S., Loudon A. (2014). Clocks for All Seasons: Unwinding the Roles and Mechanisms of Circadian and Interval Timers in the Hypothalamus and Pituitary. J. Endocrinol..

[2] Lincoln G.A., Anderson H., Loudon A. (2003). Clock Genes in Calendar Cells as the Basis of Annual Timekeeping in Mammals—A Unifying Hypothesis. J. Endocrinol..

[3] Arendt J. (2000). Melatonin, Circadian Rhythms, and Sleep. N. Engl. J. Med..

[4] Dopico X.C., Evangelou M., Ferreira R.C., Guo H., Pekalski M.L., Smyth D.J., Cooper N., Burren O.S., Fulford A.J., Hennig B.J. (2015). Widespread Seasonal Gene Expression Reveals Annual Differences in Human Immunity and Physiology. Nat. Commun..

[5] Fujisawa K., Takami T., Shintani H., Sasai N., Matsumoto T., Yamamoto N., Sakaida I. (2021). Seasonal Variations in Photoperiod Affect Hepatic Metabolism of Medaka (Oryzias Latipes). FEBS Open Bio.

[6] Togo Y., Otsuka T., Goto M., Furuse M., Yasuo S. (2012). Photoperiod Regulates Dietary Preferences and Energy Metabolism in Young Developing Fischer 344 Rats but Not in Same-Age Wistar Rats. Am. J. Physiol. Endocrinol. Metab..

[7] Chainy G.B.N., Paital B., Dandapat J. (2016). An Overview of Seasonal Changes in Oxidative Stress and Antioxidant Defence Parameters in Some Invertebrate and Vertebrate Species. Scientifica.

[8] Cruz-Carrión Á., de Azua M.J.R., Mulero M., Arola-Arnal A., Suárez M. (2020). Oxidative Stress in Rats Is Modulated by Seasonal Consumption of Sweet Cherries from Different Geographical Origins: Local vs. Non-Local. Nutrients.

[9] Rauf A., Imran M., Abu-Izneid T., Iahtisham-Ul-Haq, Patel S., Pan X., Naz S., Sanches Silva A., Saeed F., Rasul Suleria H.A. (2019). Proanthocyanidins: A Comprehensive Review. Biomed. Pharmacother..

[10] Cos P., Bruyne T., Hermans N., Apers S., Berghe D., Vlietinck A. (2012). Proanthocyanidins in Health Care: Current and New Trends. Curr. Med. Chem..

[11] Baur J.A., Sinclair D.A. (2008). What Is Xenohormesis?. Am. J. Pharmacol. Toxicol..

[12] Yokozawa T., Lee1 Y.A., Cho E.J., Matsumoto K., Park C.H., Shibahara N. (2011). Anti-Aging Effects of Oligomeric Proanthocyanidins Isolated from Persimmon Fruits. Drug Discov..

[13] Höhn A., Weber D., Jung T., Ott C., Hugo M., Kochlik B., Kehm R., König J., Grune T., Castro J.P. (2017). Happily (n)Ever after: Aging in the Context of Oxidative Stress, Proteostasis Loss and Cellular Senescence. Redox Biol..

[14] Barbosa M.C., Grosso R.A., Fader C.M. (2019). Hallmarks of Aging: An Autophagic Perspective. Front. Endocrinol..

[15] Okudan N., Barışkaner H., Gökbel H., Şahin A.S., Belviranlı M., Baysal H. (2011). The Effect of Supplementation of Grape Seed Proanthocyanidin Extract on Vascular Dysfunction in Experimental Diabetes. J. Med. Food.

[16] Ding Y., Dai X., Jiang Y., Zhang Z., Bao L., Li Y., Zhang F., Ma X., Cai X., Jing L. (2013). Grape Seed Proanthocyanidin Extracts Alleviate Oxidative Stress and ER Stress in Skeletal Muscle of Low-Dose Streptozotocin- and High-Carbohydrate/High-Fat Diet-Induced Diabetic Rats. Mol. Nutr. Food Res..

[17] Pajuelo D., Quesada H., Díaz S., Fernández-Iglesias A., Arola-Arnal A., Bladé C., Salvadó J., Arola L. (2012). Chronic Dietary Supplementation of Proanthocyanidins Corrects the Mitochondrial Dysfunction of Brown Adipose Tissue Caused by Diet-Induced Obesity in Wistar Rats. Br. J. Nutr..

[18] Fernández-Iglesias A., Pajuelo D., Quesada H., Díaz S., Bladé C., Arola L., Salvadó M.J., Mulero M. (2014). Grape Seed Proanthocyanidin Extract Improves the Hepatic Glutathione Metabolism in Obese Zucker Rats. Mol. Nutr. Food Res..

[19] Li Y., Ding Y., Zhang Z., Dai X., Jiang Y., Bao L., Li Y. (2013). Grape Seed Proanthocyanidins Ameliorate Pancreatic Beta-Cell Dysfunction and Death in Low-Dose Streptozotocin- and High-Carbohydrate/High-Fat Diet-Induced Diabetic Rats Partially by Regulating Endoplasmic Reticulum Stress. Nutr. Metab..

[20] Ibars M., Ardid-Ruiz A., Suárez M., Muguerza B., Bladé C., Aragonès G. (2016). Proanthocyanidins Potentiate Hypothalamic Leptin&sol;STAT3 Signalling and Pomc Gene Expression in Rats with Diet-Induced Obesity. Int. J. Obes..

[21] Serrano J., Casanova-Martí À., Gual A., Maria Pérez-Vendrell A., Teresa Blay M., Terra X., Ardévol A., Pinent M. (2017). A Specific Dose of Grape Seed-Derived Proanthocyanidins to Inhibit Body Weight Gain Limits Food Intake and Increases Energy Expenditure in Rats. Eur. J. Nutr..

[22] Kim K.S., Yoon Y.R., Lee H.J., Yoon S., Kim S.Y., Shin S.W., An J.J., Kim M.S., Choi S.Y., Sun W. (2010). Enhanced Hypothalamic Leptin Signaling in Mice Lacking Dopamine D2 Receptors. J. Biol. Chem..

[23] Serra A., MacI A., Romero M.P., Valls J., Bladé C., Arola L., Motilva M.J. (2010). Bioavailability of Procyanidin Dimers and Trimers and Matrix Food Effects in in Vitro and in Vivo Models. Br. J. Nutr..

[24] Smedes F., Thomasen T.K. (1996). Evaluation of the Bligh & Dyer Lipid Determination Method. Mar. Pollut. Bull..

[25] Cajka T., Fiehn O. (2015). Toward Merging Untargeted and Targeted Methods in Mass Spectrometry-Based Metabolomics and Lipidomics. Anal. Chem..

[26] Visser M.E., Caro S.P., van Oers K., Schaper S.V., Helm B. (2010). Phenology, Seasonal Timing and Circannual Rhythms: Towards a Unified Framework. Philos. Trans. R. Soc. B Biol. Sci..

[27] Ávila-Román J., Soliz-Rueda J.R., Bravo F.I., Aragonès G., Suárez M., Arola-Arnal A., Mulero M., Salvadó M.J., Arola L., Torres-Fuentes C. (2021). Phenolic Compounds and Biological Rhythms: Who Takes the Lead?. Trends Food Sci. Technol..

[28] Mariné-Casadó R., Domenech-Coca C., del Bas J.M., Bladé C., Arola L., Caimari A. (2018). The Exposure to Different Photoperiods Strongly Modulates the Glucose and Lipid Metabolisms of Normoweight Fischer 344 Rats. Front. Physiol..

[29] Patel S.R., Hu F.B. (2008). Short Sleep Duration and Weight Gain: A Systematic Review. Obesity.

[30] Heideman P.D., Sylvester C.J. (1997). Reproductive Photoresponsiveness in Unmanipulated Male Fischer 344 Laboratory Rats. Biol. Reprod..

[31] Tavolaro F.M., Thomson L.M., Ross A.W., Morgan P.J., Helfer G. (2015). Photoperiodic Effects on Seasonal Physiology, Reproductive Status and Hypothalamic Gene Expression in Young Male F344 Rats. J. Neuroendocrinol..

[32] Ross A.W., Helfer G., Russell L., Darras V.M., Morgan P.J. (2011). Thyroid Hormone Signalling Genes Are Regulated by Photoperiod in the Hypothalamus of F344 Rats. PLoS ONE.

[33] Sato M., Kanikowska D., Iwase S., Shimizu Y., Nishimura N., Inukai Y., Sato M., Sugenoya J. (2013). Seasonal Differences in Melatonin Concentrations and Heart Rates during Sleep in Obese Subjects in Japan. Int. J. Biometeorol..

[34] Spady D.K., Woollett L.A., Dietschy J.M. (1993). Regulation of Plasma LDL-Cholesterol Levels by Dietary Cholesterol and Fatty Acids. Annu. Rev. Nutr..

[35] Sun D.-Q., Liu W.-Y., Wu S.-J., Zhu G.-Q., Braddock M., Zhang D.-C., Shi K.-Q., Song D., Zheng M.-H., Sun D.-Q. (2015). Increased Levels of Low-Density Lipoprotein Cholesterol within the Normal Range as a Risk Factor for Nonalcoholic Fatty Liver Disease. Oncotarget.

[36] Sasaki J., Kumagae G., Sata T., Ikeda M., Tsutsumi S., Arakawa K. (1983). Seasonal Variation of Serum High Density Lipoprotein Cholesterol Levels in Men. Atherosclerosis.

[37] Fager G., Wiklund O., Olofsson S.O., Bondjers G. (1982). Seasonal Variations in Serum Lipid and Apolipoprotein Levels Evaluated by Periodic Regression Analyses. J. Chronic Dis..

[38] Ma Y., Olendzki B.C., Li W., Hafner A.R., Chiriboga D., Hebert J.R., Campbell M., Sarnie M., Ockene I.S. (2006). Seasonal Variation in Food Intake, Physical Activity, and Body Weight in a Predominantly Overweight Population. Eur. J. Clin. Nutr..

[39] Carabaza A., Ciudad C.J., Baqué S., Guinovart J.J. (1992). Glucose Has to Be Phosphorylated to Activate Glycogen Synthase, but Not to Inactivate Glycogen Phosphorylase in Hepatocytes. FEBS Lett..

[40] Nakamura T., Kato S., Ichihara A. (1984). Glucagon and Glucose as Major Regulators of Glycogen Metabolism in Primary Cultured Rat Hepatocytes. J. Biochem..

[41] Stalmans W., de Wulf H., Hue L., Hers H.-G. (1974). The Sequential Inactivation of Glycogen Phosphorylase and Activation of Glycogen Synthetase in Liver after the Administration of Glucose to Mice and Rats. The Mechanism of the Hepatic Threshold to Glucose. Eur. J. Biochem..

[42] Gumaa K.A., MacLeod R.M., McLean P. (1969). The Pentose Phosphate Pathway of Glucose Metabolism. Influence of a Growth-Hormone-Secreting Pituitary Tumour on the Oxidative and Non-Oxidative Reactions of the Cycle in Liver. Biochem. J..

[43] Jin E.S., Sherry A.D., Malloy C.R. (2014). Interaction between the Pentose Phosphate Pathway and Gluconeogenesis from Glycerol in the Liver. J. Biol. Chem..

[44] Rockall A.G., Sohaib S.A., Evans D., Kaltsas G., Isidori A.M., Monson J.P., Besser G.M., Grossman A.B., Reznek R.H. (2003). Hepatic Steatosis in Cushing’s Syndrome: A Radiological Assessment Using Computed Tomography. Eur. J. Endocrinol..

[45] Amatruda J.M., Livingston J.N., Lockwood D.H. (1985). Cellular Mechanisms in Selected States of Insulin Resistance: Human Obesity, Glucocorticoid Excess, and Chronic Renal Failure. Diabetes/Metab. Rev..

[46] Oleshchuk O., Ivankiv Y., Falfushynska H., Mudra A., Lisnychuk N. (2019). Hepatoprotective Effect of Melatonin in Toxic Liver Injury in Rats. Medicina.

[47] Bonomini F., Borsani E., Favero G., Rodella L.F., Rezzani R. (2018). Dietary Melatonin Supplementation Could Be a Promising Preventing/Therapeutic Approach for a Variety of Liver Diseases. Nutrients.

[48] Zhang J.-J., Meng X., Li Y., Zhou Y., Xu D.-P., Li S., Li H.-B. (2017). Molecular Sciences Effects of Melatonin on Liver Injuries and Diseases. Int. J. Mol. Sci..

[49] Ohta Y., Kongo M., Sasaki E., Nishida K., Ishiguro I. (2000). Therapeutic Effect of Melatonin on Carbon Tetrachloride-Induced Acute Liver Injury in Rats. J. Pineal Res..

[50] Tahan G., Akin H., Aydogan F., Ramadan S.S., Yapicier O., Tarcin O., Uzun H., Tahan V., Zengin K. (2010). Melatonin Ameliorates Liver Fibrosis Induced by Bile-Duct Ligation in Rats. Can. J. Surg..

[51] Chojnacki C., Walecka-Kapica E., Romanowski M., Chojnacki J., Klupinska G. (2014). Protective Role of Melatonin in Liver Damage. Curr. Pharm. Des..

[52] Ribas-Latre A., del Bas J.M., Baselga-Escudero L., Casanova E., Arola-Arnal A., Salvadó M.J., Arola L., Bladé C. (2015). Dietary Proanthocyanidins Modulate Melatonin Levels in Plasma and the Expression Pattern of Clock Genes in the Hypothalamus of Rats. Mol. Nutr. Food Res..

[53] Basseri S., Austin R.C. (2008). ER Stress and Lipogenesis: A Slippery Slope toward Hepatic Steatosis. Dev. Cell.

[54] Colgan S.M., Tang D., Werstuck G.H., Austin R.C. (2007). Endoplasmic Reticulum Stress Causes the Activation of Sterol Regulatory Element Binding Protein-2. Int. J. Biochem. Cell Biol..

[55] Lee J.N., Ye J. (2004). Proteolytic Activation of Sterol Regulatory Element-Binding Protein Induced by Cellular Stress through Depletion of Insig-1. J. Biol. Chem..

[56] Kim Y.R., Lee E.J., Shin K.O., Kim M.H., Pewzner-Jung Y., Lee Y.M., Park J.W., Futerman A.H., Park W.J. (2019). Hepatic Triglyceride Accumulation via Endoplasmic Reticulum Stress-Induced SREBP-1 Activation Is Regulated by Ceramide Synthases. Exp. Mol. Med..

[57] Kim D.S., Jeong S.K., Kim H.R., Kim D.S., Chae S.W., Chae H.J. (2007). Effects of Triglyceride on ER Stress and Insulin Resistance. Biochem. Biophys. Res. Commun..

[58] Wei Y., Wang D., Topczewski F., Pagliassotti M.J. (2006). Saturated Fatty Acids Induce Endoplasmic Reticulum Stress and Apoptosis Independently of Ceramide in Liver Cells. Am. J. Physiol. Endocrinol. Metab..

[59] Ding R.-B., Bao J., Deng C.-X. (2017). Emerging Roles of SIRT1 in Fatty Liver Diseases. Int. J. Biol. Sci.

[60] Kim H.S., Xiao C., Wang R.H., Lahusen T., Xu X., Vassilopoulos A., Vazquez-Ortiz G., Jeong W.-I., Park O., Ki S.H. (2010). Hepatic Specific Disruption of SIRT6 in Mice Results in Fatty Liver Formation Due to Enhanced Glycolysis and Triglyceride Synthesis. Cell Metab..

[61] Aragonès G., Suárez M., Ardid-Ruiz A., Vinaixa M., Rodríguez M.A., Correig X., Arola L., Bladé C. (2016). Dietary Proanthocyanidins Boost Hepatic NAD + Metabolism and SIRT1 Expression and Activity in a Dose-Dependent Manner in Healthy Rats. Sci. Rep..

[62] Foretz M., Guichard C., Ferré P., Foufelle F. (1999). Sterol Regulatory Element Binding Protein-1c Is a Major Mediator of Insulin Action on the Hepatic Expression of Glucokinase and Lipogenesis-Related Genes. Proc. Natl. Acad. Sci. USA.

[63] Dentin R., Pé J.-P., Benhamed F., Foufelle F., Ferré P., Ronique Fauveau V., Magnuson M.A., Girard J., Postic C. (2004). Hepatic Glucokinase Is Required for the Synergistic Action of ChREBP and SREBP-1c on Glycolytic and Lipogenic Gene Expression. J. Biol. Chem..

[64] Ikegami K., Iigo M., Yoshimura T. (2013). Circadian Clock Gene Per2 Is Not Necessary for the Photoperiodic Response in Mice. PLoS ONE.

[65] Lamia K.A., Storch K.-F., Weitz C.J. (2008). Physiological Significance of a Peripheral Tissue Circadian Clock. Proc. Natl. Acad. Sci. USA.

[66] Xie X., Zhao B., Huang L., Shen Q., Ma L., Chen Y., Wu T., Fu Z. (2017). Effects of Altered Photoperiod on Circadian Clock and Lipid Metabolism in Rats. Chronobiol. Int..

