# Supplementary material for: Grape-Seed Procyanidin Extract (GSPE) Seasonal-Dependent Modulation of Glucose and Lipid Metabolism in the Liver of Healthy F344 Rats
Source: Biomolecules. 2022 Jun 17;12(6):839. doi: 10.3390/biom12060839 (PMC9221469; doi:10.3390/biom12060839)
Supplement: Supplementary file 1 [file biomolecules-12-00839-s001.zip › biomolecules-1749635-supplementary.pdf]

Table S1. Treatment comparation between metabolites related to hepatic glucose metabolism. Metabolites of glucose metabolism in the liver of Fischer 344 rats fed a STD diet and exposed to three different photoperiods for 9 weeks, supplemented with vehicle or GSPE for the last 4 weeks. The statistically significant p-values ($p < 0.05$) are highlighted in bold.

Metabolites	L12				L18				L6			
	STD-VH	STD-GSPE	p-value	FC	STD-VH	STD-GSPE	p-value	FC	STD-VH	STD-GSPE	p-value	FC
Pyruvic acid	0.62 ± 0.05	0.55 ± 0.07	0.424	0.9	0.87 ± 0.19	0.56 ± 0.05	0.144	0.6	0.68 ± 0.09	0.62 ± 0.07	0.661	0.9
Lactic acid	20.07 ± 0.74	20.01 ± 0.89	0.957	1	20.66 ± 1.78	20.34 ± 0.7	0.871	1	21.52 ± 1.21	19.21 ± 0.86	0.141	0.9
Glycerol	0.08 ± 0.01	0.07 ± 0.01	0.407	0.8	0.07 ± 0.01	0.06 ± 0	0.218	0.8	0.08 ± 0.01	0.07 ± 0.01	0.568	0.9
Glycerol-1-phosphate	1.6 ± 0.09	1.51 ± 0.08	0.421	0.9	1.53 ± 0.09	1.62 ± 0.09	0.492	1.1	1.69 ± 0.12	1.56 ± 0.1	0.404	0.9
d-Glucose	150.4 ± 2.92	150.72 ± 2.33	0.933	1	156.07 ± 2.94	147.73 ± 1.19	**0.027**	0.9	154.73 ± 4.04	151.43 ± 2.78	0.511	1
d-Ribose	0.02 ± 0	0.01 ± 0	0.582	0.9	0.05 ± 0.02	0.01 ± 0	**0.027**	0.2	0.02 ± 0	0.01 ± 0	0.187	0.7
Ribose-5-phosphate	0.62 ± 0.05	0.55 ± 0.07	0.424	0.9	0.87 ± 0.19	0.56 ± 0.05	0.144	0.6	0.68 ± 0.09	0.62 ± 0.07	0.661	0.9
Fructose-6-phosphate	0.03 ± 0	0.02 ± 0	**0.018**	0.6	0.03 ± 0.01	0.04 ± 0.01	0.37	1.3	0.03 ± 0	0.03 ± 0	0.418	1.2
Glucose-6-phosphate	0.08 ± 0.01	0.05 ± 0.01	**0.033**	0.6	0.07 ± 0.01	0.09 ± 0.01	0.318	1.3	0.06 ± 0.01	0.08 ± 0.01	0.44	1.2

Table S2. Photoperiod comparation between metabolites related to hepatic glucose metabolism. Metabolites of glucose metabolism in the liver of Fischer 344 rats fed a STD diet and exposed to three different photoperiods for 9 weeks, supplemented with vehicle or GSPE for the last 4 weeks. The statistically significant p-values ($p < 0.05$) are highlighted in bold.

Metabolites	L12-VH vs. L18-VH		L12- VH vs. L6-VH		L18-VH vs. L6-VH		L12-GSPE vs. L18-GSPE		L12-GSPE vs. L6-GSPE		L18-GSPE vs. L6-GSPE	
	p-value	FC	p-value	FC	p-value	FC	p-value	FC	p-value	FC	p-value	FC
Pyruvic acid	0.241	1.4	0.639	1.1	0.381	0.8	0.931	1	0.472	1.1	0.47	1.1
Lactic acid	0.766	1	0.323	1.1	0.692	1	0.775	1	0.527	1	0.325	0.9
Glycerol	0.564	0.9	0.876	1	0.652	1.1	0.469	0.9	0.899	1	0.417	1.2
Glycerol-1-phosphate	0.576	1	0.549	1.1	0.298	1.1	0.356	1.1	0.664	1	0.655	1
d-Glucose	0.193	1	0.399	1	0.793	1	0.295	1	0.848	1	0.267	1
d-Ribose	**0.043**	3.1	0.308	1.4	0.102	0.4	0.159	0.7	0.947	1	0.12	1.5
Ribose-5-phosphate	0.241	1.4	0.639	1.1	0.381	0.8	0.931	1	0.472	1.1	0.47	1.1
Fructose-6-phosphate	0.411	0.8	0.266	0.8	0.87	1	**0.038**	1.7	0.055	1.5	0.661	0.9
Glucose-6-phosphate	0.432	0.8	0.269	0.8	0.791	0.9	0.043	1.7	0.095	1.5	0.506	0.9

Table S3: Summary of the main metabolic changes due to the GSPE treatment in each photoperiod compared to its respective VH control.

pathway/treatment	GSPE L12	GSPE L18	GSPE L6
GLUCONEOGENESIS	equal	equal	increased
GLUCOLISIS	equal	increased	equal
LIPOLISIS	equal	equal	equal
LIPOGENESIS	equal	decreased	equal

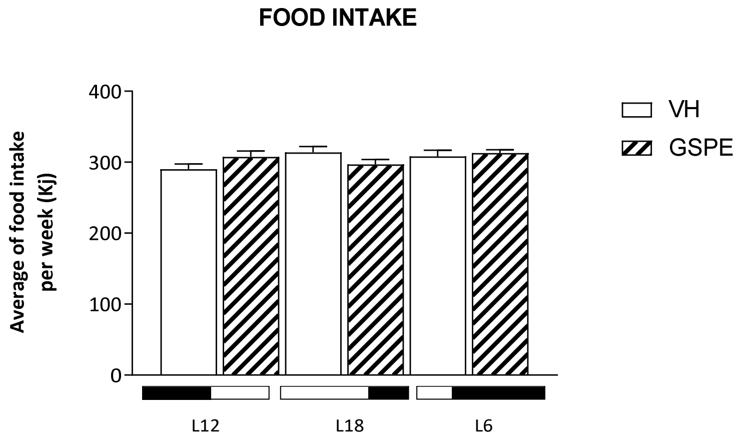

Figure S1: Average of weekly food intake (kJ) of different photoperiods
